# CD4 T-Cell Subsets in Malaria: TH1/TH2 Revisited

**DOI:** 10.3389/fimmu.2014.00671

**Published:** 2015-01-12

**Authors:** Damian Perez-Mazliah, Jean Langhorne

**Affiliations:** ^1^Division of Parasitology, MRC National Institute for Medical Research, London, UK

**Keywords:** malaria, *Plasmodium*, multifunctional CD4 T-cells, CD4 T-cell subsets, Tfh, Th1, Th2, Th22

## Abstract

CD4^+^ T-cells have been shown to play a central role in immune control of infection with *Plasmodium* parasites. At the erythrocytic stage of infection, IFN-γ production by CD4^+^ T-cells and CD4^+^ T-cell help for the B-cell response are required for control and elimination of infected red blood cells. CD4^+^ T-cells are also important for controlling *Plasmodium* pre-erythrocytic stages through the activation of parasite-specific CD8^+^ T-cells. However, excessive inflammatory responses triggered by the infection have been shown to drive pathology. Early classical experiments demonstrated a biphasic CD4^+^ T-cell response against erythrocytic stages in mice, in which T helper (Th)1 and antibody-helper CD4^+^ T-cells appear sequentially during a primary infection. While IFN-γ-producing Th1 cells do play a role in controlling acute infections, and they contribute to acute erythrocytic-stage pathology, it became apparent that a classical Th2 response producing IL-4 is not a critical feature of the CD4^+^ T-cell response during the chronic phase of infection. Rather, effective CD4^+^ T-cell help for B-cells, which can occur in the absence of IL-4, is required to control chronic parasitemia. IL-10, important to counterbalance inflammation and associated with protection from inflammatory-mediated severe malaria in both humans and experimental models, was originally considered be produced by CD4^+^ Th2 cells during infection. We review the interpretations of CD4^+^ T-cell responses during *Plasmodium* infection, proposed under the original Th1/Th2 paradigm, in light of more recent advances, including the identification of multifunctional T-cells such as Th1 cells co-expressing IFN-γ and IL-10, the identification of follicular helper T-cells (Tfh) as the predominant CD4^+^ T helper subset for B-cells, and the recognition of inherent plasticity in the fates of different CD4^+^ T-cells.

## Introduction

**Figure 1:**
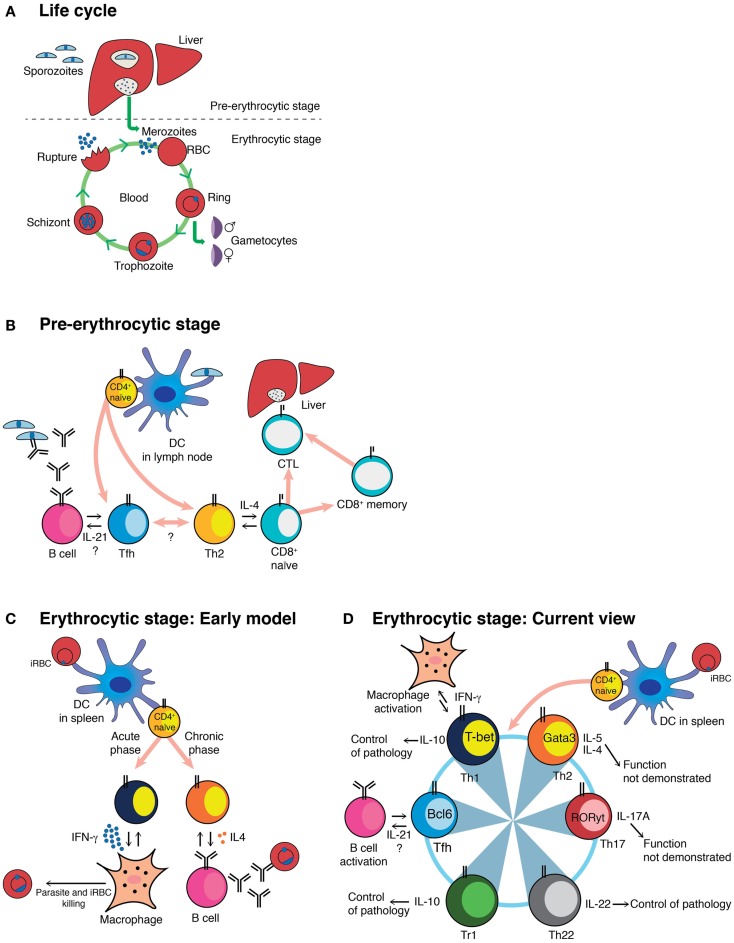
**Schematic representation of the *Plasmodium* life cycle, and different models of CD4^+^ T-cell activation during *Plasmodium* infection**. **(A)** *Plasmodium* life cycle in the mammalian host. **(B)** The cartoon shows the different subsets known, or proposed to be, activated by the pre-erythrocytic stage of *Plasmodium*, together with their known or proposed functions. **(C)** Classical view of the biphasic activation of Th1 and Th2 CD4^+^ T-cells toward the erythrocytic stage of *Plasmodium*. **(D)** Current understanding of the CD4^+^ T-cell responses to the erythrocytic stage of *Plasmodium*, together with their known or proposed roles during infection. This model highlights the plastic ability of activated CD4^+^ T-cells to interconvert into different Th subsets. The master regulator transcription factors known to drive each Th program as well as the cytokines associated to each Th subset are depicted (53). DC, myeloid dendritic cell; iRBC, infected red blood cell; CTL, cytotoxic CD8^+^ T-cell; Tr1, Foxp3^−^ regulatory T-cell.

Malaria, caused by infection with *Plasmodium* transmitted via mosquito bites, represents a major global cause of morbidity and mortality (1). *Plasmodium* spp. are eukaryotic apicomplexan intracellular parasites with different life-cycle stages within the vertebrate host: an early clinically silent liver stage that can last approximately 7–10 days in humans and 2 days in rodents, followed by an erythrocytic stage, responsible for the pathology of malaria (Figure 1A). Species of *Plasmodium* that infect humans include *P. falciparum, P. vivax, P. malariae, P. ovale*, and *P. knowlesi*. A number of *Plasmodium* species that infect rodents, but not humans, are available for laboratory research, including *P. berghei, P. vinckei, P. chabaudi*, and *P. yoelii* (2), which allow the dissection of immune mechanism of protection and pathology (3).

In most cases, the host’s immune system can eventually control a *Plasmodium* infection; however, exacerbated host immune responses and inflammation induced by the parasite, contribute to the pathology accompanying infection (4, 5). CD4^+^ T-cell responses have been associated with control of erythrocytic stage parasites, but a small number of studies indicate a helper role also in pre-erythrocytic immunity (6–11). Parasite biology, host cell and tissue tropism, and kinetics of parasite growth differ between pre-erythrocytic and erythrocytic stages within the vertebrate host and, accordingly, the particular CD4^+^ T-cell responses elicited also differ. Herein, we discuss activation of different CD4^+^ T-cell subsets during malaria, their role in the control of the infection and the interplay between different subsets, with a particular emphasis on the concept of CD4^+^ T-cell plasticity.

## CD4^+^ T-Cell Subsets Activated by Pre-Erythrocytic Stages

Very little is known about the CD4^+^ T-cell response to *Plasmodium* pre-erythrocytic stages, or its regulation in natural infection either in humans or in experimental models. Clearly, since IgG antibodies and memory B-cells are generated to a wide range of pre-erythrocytic antigens, including those with expression restricted primarily to these stages, such as circumsporozoite protein (CSP), liver-stage antigen 1 (LSA1), and sporozoite threonine–asparagine-rich protein (STARP) (12–14), CD4^+^ T-cells must be induced by these stages of the infection. Indeed, CD4^+^ T-cells specific for pre-erythrocytic antigens have been documented, and in some cases, have been shown to correlate with protection in humans following natural infection (15) and immunization (11). However, we have few details of their functional heterogeneity.

CD4^+^ T-cells of undefined Th1/Th2 phenotype have been shown to confer protection against the pre-erythrocytic stages of *P. yoelii* even in the absence of CD8^+^ T-cells (9), and CD4^+^ T-cell clones recognizing peptides of CSP protected against a *P. yoelii* sporozoite challenge in mice, irrespective of their Th1 or Th2 phenotype (6, 7). The location of priming of CD4^+^ T-cell specific for pre-erythrocytic stages is still a matter of debate, and there is little evidence as yet on priming of CD4^+^ T-cell in the liver. As protective CD8^+^ T-cells specific for a peptide of CSP from *P. yoelii* can be primed by dendritic cells (DCs) in lymph nodes after infection with sporozoites (16), it is likely that DCs in lymph nodes might also be critical for priming CD4^+^ T-cell responses to *Plasmodium* pre-erythrocytic stages.

As well as possible direct killing of infected hepatocytes, subsets of CD4^+^ T-cells provide crucial help for both B-cell and CD8^+^ T-cell responses (Figure 1B). In a very few studies, CD4^+^ T-cells were shown to be necessary to ensure survival of protective effector and memory CD8^+^ T-cells induced by irradiation-attenuated sporozoites (8, 10). This mechanism is dependent on STAT-6 and IL-4, suggesting that Th2 CD4^+^ T-cells may be in charge of providing help to CD8^+^ T-cells. More recently, CSP-specific CD4^+^ T-cells expressing CD107a (LAMP-1), a marker for cytotoxic degranulation, were shown to be induced and associated with protection against the pre-erythrocytic stages after immunizations of healthy volunteers by bites from *P. falciparum*-infected mosquitoes during chloroquine chemoprophylaxis (11). Nothing is yet known about induction of Tfh cells, the subset that provides help for T-cell dependent B-cell responses (17), which are presumably activated by, and would be important for, generation of high affinity IgG antibodies.

Altogether, these data suggest a role of CD4^+^ T-cells in protective immunity to pre-erythrocytic-stage infection, the mechanisms of which are not yet completely understood. With the current emphasis on pre-erythrocytic vaccines, it is important that we understand more about the potential contribution of diverse CD4^+^ T-cell populations on direct killing of infected cells, in the B-cell and CD8^+^ T-cell responses, as well as their regulatory roles in immunity to the pre-erythrocytic stages of *Plasmodium*.

## CD4^+^ T-Cell Subsets Activated by Erythrocytic Stages

The Th1/Th2 paradigm proposed by Mosmann and Coffman postulated stable lineages of activated CD4^+^ T-cells with distinctive cytokine production patterns and functional capacity; IFN-γ-producing Th1 cells being crucial mediators of host immunity against intracellular pathogens, while IL-4-producing Th2 cells mediating immunity toward extracellular pathogens and collaboration with B-cells for antibody production (18). As *Plasmodium* invades red blood cells (Figure 1A), which do not express MHC class I or II, it was difficult to envisage parasites at this stage as direct targets of Th1 or Th2 cells. Nonetheless, it was possible to draw parallels with the original Mosmann and Coffman model; malaria researchers observed that the erythrocytic stages triggered a strong IFN-γ response during acute infections in *P. berghei, P. yoelii*, and *P. chabaudi* infections in mice, as well as in *P. falciparum* infection in humans (19–24).

Since that time, the Th1/Th2 paradigm has gained in complexity with the identification of novel CD4^+^ T-cell subsets with distinctive characteristics and transcriptional programs in charge of driving the different cell fates (25). These differentiation programs are governed predominantly by signals derived from antigen-presenting cells (APC) and the microenvironment at the time of CD4^+^ T-cell activation. DCs are necessary for effective priming of the T-cell response in erythrocytic-stage malaria (26), and two subsets of splenic DCs, CD8^−^ and CD4^+^ classical DCs, have been shown to present antigen for the activation of CD4^+^ T-cells during an erythrocytic-stage infection with *P. chabaudi* and *P. berghei*, respectively (26–29). Although it is known that IL-12 is an important cytokine in the induction of a protective response in experimental malaria infections (30), understanding of the regulation of this cytokine or other factors in DCs necessary for effective *Plasmodium*-antigen presentation to different subsets of CD4^+^ T-cells is still lacking.

IFN-γ, a defining cytokine of Th1 cells expressing the transcription factor T-bet, has proven to be important for controlling the acute erythrocytic stage of *Plasmodium* infection in rodent models (31–34). IFN-γ-producing CD4^+^ effector (E) and effector memory (EM) CD4^+^ T-cells both confer partial protection from *P. chabaudi* infection (35). In general agreement with this, IFN-γ from CD4^+^ T-cells has been shown to be important in maintaining strain-transcending blood-stage immunity (36). However, IFN-γ is not only produced by T-bet^+^ Th1 cells but also by NK cells, NKT cells, and γδ T-cells (37, 38) as well as CD8^+^ T-cells, and it is not always clear whether Th1 cells, IFN-γ, or IFN-γ from Th1 cells *per se* are the main players in early protection or pathology in experimental malaria. Studies so far to address these questions have given conflicting results. One study using *P. berghei* ANKA has shown that the enhanced IFNγ^+^ T-bet^+^ CD4^+^ T-cell responses observed in mice lacking Type I IFN signaling are associated with better control of *P. berghei* ANKA infections, resulting in lower morbidity and mortality (39, 40). In contrast, others have shown that in the absence of T-bet, essential for Th1 commitment, cerebral pathology of *P. berghei* ANKA infections is ameliorated, and the number of IFNγ^+^ CD4^+^ T-cells is reduced. However, control of parasite replication is lost and mice succumb to hyper-parasitemia and anemia (41). In a different rodent model of erythrocytic-stage malaria, *P. yoelii* 17X(NL), although activation of T-bet was detected on CD4^+^ T-cells early in infection (42), the infection can still be controlled in T-bet-deficient mice (43).

The erythrocytic stages of *Plasmodium* are also able to activate CD4^+^ T-cells that are very effective helpers for *Plasmodium*-specific antibody production, but produce little or no IFN-γ (21). This response was shown to coincide with the appearance of IL-4-producing CD4^+^ T-cells (21). The association between IL-4-producing CD4^+^ T-cells and antibody responses toward the parasite was also observed in *P. falciparum* immune subjects (44). An erythrocytic-stage *P. chabaudi* infection, the only mouse model that generates a chronic phase of infection (3), presents a biphasic CD4^+^ T-cell activation, with a large IFN-γ-producing CD4^+^ T-cell response during the acute phase, followed by an antibody-helper/IL-4-producing CD4^+^ T-cell response during the chronic phase (Figure 1C) (21, 24, 45, 46). These data were interpreted as an early activation of Th1 cells able to control parasitemia through the activation of effector mechanisms such as macrophages, followed by a Th2 response in charge of activating B-cell responses to complete the clearance of the parasite (47, 48). However, the frequency of CD4^+^ T-cells able to help B-cells to produce *Plasmodium*-specific antibodies was much higher than the frequency of IL-4-producing CD4^+^ T-cells (21). Furthermore, control of a *P. chabaudi* infection and specific IgG responses, including IgG1 antibodies, was possible even in the complete absence of IL-4 (45). Therefore, it was clear, despite its attractiveness as a model, that the simple Th1/Th2 paradigm was not sufficient to explain the full complexity of CD4^+^ T-cell activation in the erythrocytic stages of *Plasmodium*. More recently, a subset of CD4^+^ T-cells, Tfh cells, has been described that produce IL-21, as well as other cytokines originally associated with other Th subsets, such as IFN-γ and IL-4 (17). We believe that the Tfh program, and not a Th2 response, is the critical one for B-cell help and activation of protective B-cell responses against the erythrocytic stages of *Plasmodium* infection (Figure 1D). However, there are very few data on Tfh or its crucial signature cytokine, IL-21, in malaria. Lymphocytes closely resembling Tfh have been observed in peripheral blood of humans (49), although not yet in people exposed to malaria. However, IL-21-producing CD4^+^ T-cells have been demonstrated in blood of immune adults living in endemic areas of *P. falciparum* transmission (50–52). Given the importance of the humoral response in protective immunity to the erythrocytic stages of *Plasmodium*, understanding the activation and maintenance of Tfh cells during malaria is of outstanding interest for vaccine design.

Recently, the Th17 subset of CD4^+^ T-cells, defined by the expression of the transcription factor RORγt, has gained attention among malaria researchers because of its role in autoimmune diseases and chronic inflammation and in responses to extracellular pathogens such as bacteria and fungi (53). CD4^+^ IL-17A^+^ RORγt^+^ Th17 cells are activated during acute *P. berghei ANKA* and *P. yoelii* infection, but the function of these cells during infection was not explored (54). Ishida and colleagues demonstrated no association of Th17 cells and cerebral malaria in *P. berghei ANKA*-infected IL-17-deficient mice (55). We have also observed the presence of IL-17A and IL-17F-producing CD4^+^ T-cells mainly in the liver during acute erythrocytic-stage *P. chabaudi* infection; however, IL-17A-deficient mice showed no significant alterations in the course of *P. chabaudi* infection (56). Therefore, despite activation, Th17 cells have so far not been shown to have a defined role during *Plasmodium* infections (Figure 1D).

Additional CD4^+^ T-cell subsets, such as that producing IL-22 (Th22), continue to be identified (57, 58). IL-22 has been implicated in both host defense against bacterial infections and tissue repair (59). Interestingly, IL-22 single-nucleotide polymorphisms associated with resistance and susceptibility to severe malaria have been identified (60). We have observed that IL-22-producing CD4^+^ T-cells are activated, albeit in low frequency, in both spleen and liver during acute erythrocytic-stage *P. chabaudi* infections, and that IL-22-deficient mice infected with *P. chabaudi* show exacerbated pathology (56) (Figure 1D). Research is ongoing to explore in greater detail the origins of these cells, their location, and the mechanisms underlying the observed pathology.

With the discovery of this wide array of possible CD4^+^ T-cell subsets and their different activation requirements and functional capacities, it is becoming clear that CD4^+^ T-cells may not be simply defined as individual subsets of Th cells producing a single cytokine, but rather they represent components of a dynamic and interactive response, in which these cells can be multifunctional, flexible, and plastic depending on the disease/infection and activation environment (61). The multifunctional capacity of T-cells, or the ability to perform more than one function (e.g., production of different cytokines) at the single-cell level, and its association with the capacity to control infections was first recognized in HIV-infected subjects (62) and in a mouse model of vaccination against *Leishmania major* (63). This association between multifunctional capacity of T-cells and control of infections is not limited to HIV and *Leishmania*, and was soon observed in several other chronic infections, including viral, parasitic, and mycobacterial infections (64). Immunization of subjects with *P. falciparum* apical membrane antigen 1 (AMA1) (65), and immunizations of mice with full-length *P. falciparum* CSP protein (66) also activates multifunctional CD4^+^ T-cells responses. Moreover, multi-parameter flow cytometric analyses of human PBMC from children and adults exposed to malaria infection reveal the existence of CD4^+^ T-cells co-expressing several cytokines characteristic of many CD4^+^ T-cell subsets (52, 67), demonstrating the complexity of the CD4^+^ T-cell response activated by the erythrocytic stages of *Plasmodium*.

There is an important body of evidence suggesting that, far from being terminally differentiated stable lineages, the different Th subsets have an extensive capacity to interconvert further between different phenotypes, a concept known as plasticity (68). The most recent studies suggest that CD4^+^ T-cell activation with overlapping characteristics of different Th subsets is the norm rather than the exception (61), and this is likely to be reflected in complex diseases such as malaria. Although this has not been explored in any detail in the context of malaria, the concept of Th plasticity opens new possibilities for studying the function and regulation of CD4^+^ T-cells in the control of *Plasmodium* infection and related immunopathology. One subset known for its remarkable plasticity is the Tfh subset. It has been shown that Th1, Th2, and Th17 cells can migrate into the B-cell areas of secondary lymphoid organs and acquire the functional capacity and biomarkers of Tfh cells and, conversely, the Tfh subset can become Th1, Th2, and Th17 (69). In the context of *Plasmodium* infection, this would imply that not only could *Plasmodium* parasites activate Tfh responses directly but also the Tfh subset could potentially arise from any of the CD4^+^ T-cell subsets already activated by the erythrocytic stages.

In light of the multifunctional and plastic capacities of the CD4^+^ T-cells, a scenario can be envisaged in which Th subsets required for the control of parasite burden, such as Th1 cells, have the capacity to acquire a regulatory phenotype depending on the context, contributing to control of the inflammation and thus to protection of tissues and organs and preventing any potentially harmful effects of the response. This would allow a fine-tuning of the CD4^+^ T-cell response to guarantee the control of *Plasmodium* infection without causing deleterious side effects. One mechanism of self-regulation by CD4^+^ Th1 cells in malaria is the induction of IL-10. IFN-γ^+^IL-10^+^T-bet^+^ Th1 CD4^+^ T-cells can prevent pathology during *P. chabaudi* infection (70) (Figure 1D). In addition, IL-10 from CD4^+^ T-cells distinct from regulatory T (Treg) cells is able to control pathology in a *P. yoelii* infection in mice, but in this case, these cells do not co-express IFN-γ (71). IL-10 is produced in these cells in response to IL-27 (70), although the signals responsible for the induction of IL-27 remain unknown. The occurrence of IFN-γ^+^IL-10^+^T-bet^+^ CD4^+^ T-cells during *Plasmodium* infections is not restricted to mouse models; they have been reported to be present in PBMC of children living in highly malaria-endemic regions (72–74) and their proportion is higher in children with uncomplicated malaria compared to children with severe malaria (72). IL-10 can also be induced in IL-17-producing CD4^+^ T-cells, as yet by unknown pathways (75–77), and thus, IL-10 may be a more general mechanism for regulating any subset of CD4^+^ T-cells in malaria. CD4^+^ T-cells, particularly Th1 cells, can also be controlled by Type I IFNs. In the *P. berghei* ANKA model, Type I IFN signaling suppresses Th1 responses by directly acting on classical DCs (40). Given that type I IFN signaling can also promote the expression of IL-10 on CD4^+^ T-cells (78–81), we hypothesize that these two regulatory mechanisms might share some common activation signals during *Plasmodium* infection.

CD4^+^ T-cell responses may also be controlled by the expression of surface molecules associated with exhaustion. Elevated frequencies of PD-1^+^ LAG-3^+^ CD4^+^ T-cells have been reported in *P. falciparum*-infected subjects (82, 83), and combined blockade of PD-1 and LAG-3 accelerated clearance of erythrocytic-stage *Plasmodium* infection in a mouse model (83). In agreement with these observations, PD-1-deficient mice show better control of an erythrocytic-stage *P. chabaudi* infection with higher frequencies of IFN-γ^+^ and T-bet^+^ CD4^+^ T-cells during the chronic phase (84). The kinetics of PD-1^+^ CD4^+^ T-cells during the acute erythrocytic-stage *P. yoelii* 17X(NL) infection are similar to those observed during *P. chabaudi* infection (42, 84). However, some caution should be exercised in assuming that expression of PD-1 automatically means exhaustion, as in some subsets of activated CD4^+^ T-cells, in particular Tfh cells, PD1 is expressed without affecting their functional capacity. It may be that the triggering of PD-1 by its ligand PDL-1 (85) is the key to whether the cell is programed for cell death.

Many of the CD4^+^ T-cells activated in a *Plasmodium* infection may undergo interconversion between defined cell subsets depending on antigen dose, APC, location, and cytokine/chemokine environment, such as that described for Treg and Th17 subsets (86, 87). Thus, it is possible that the Th17 cells found in the spleen of malaria-infected mice gain a regulatory phenotype in other organs or tissues such as brain and liver. The capacity to identify and manipulate these possible mechanisms of CD4^+^ T-cell plasticity during *Plasmodium* infections would be of great value for the design of novel therapeutic strategies.

## Concluding Remarks

The identification of two CD4^+^ T-cell subsets with different well-defined functions represented an attractive organizational system with which to rationalize CD4^+^ T-cell responses to *Plasmodium* infections. However, such a model has not been sufficient to reflect fully the complexity of CD4^+^ T-cell biology observed in human or experimental malaria. In particular, control of *Plasmodium* infection requires strictly regulated immune responses that are able to prevent parasite replication without causing detrimental side effects of uncontrolled inflammation. *Plasmodium* species have different stages with different tissue tropisms and this complex life cycle challenges the idea that a single static group of terminally differentiated CD4^+^ T-cells would be able to perform all the tasks required to control this infection. In order to cope with these tasks, the CD4^+^ T-cell response has to adapt to the changing scenarios presented as the infection evolves. The newer concept of CD4^+^ T-cell plasticity would add substantially to our understanding of induction and regulation of CD4^+^ T-cell responses in malaria, and it is highly probable that some of the CD4^+^ Th programs not yet explored in depth, such as the Tfh response, might play critical roles in the outcome of the infection. The combination of potent tools such as multi-parameter flow cytometry, *in vivo* imaging, systems analyses of transcriptome, proteome, and metabolome, together with T-cell receptor transgenic mice and peptide-MHC II tetramers will give us the chance to explore the complexity of the CD4^+^ T-cell responses to malaria in *in vivo* models (74, 88–97). In addition, confocal microscopy and intravital imaging techniques make now possible to follow sporozoites injected via the mosquito bite into the skin, or by injection of attenuated sporozoites through to their arrival in the lymphoid organs and liver (98), and to study the consequent activation of CD4^+^ T-cells and their subsequent effector functions. Field studies of natural human *Plasmodium* infections and mouse models should complement each other to get a deeper understanding of the complex CD4^+^ T-cell response activated by these infections. A detailed delineation on how CD4^+^ T-cells modulate the activation of effector cells such as CD8^+^ T-cells, macrophages, and B-cells in response to *Plasmodium* infection is critical to achieve the goal of generating protective treatments to control malaria.

## Conflict of Interest Statement

The authors declare that the research was conducted in the absence of any commercial or financial relationships that could be construed as a potential conflict of interest.
